# The moderating effects of perceived transportation access on health and social connectedness for people with disabilities

**DOI:** 10.1016/j.jth.2025.102165

**Published:** 2025

**Authors:** Aaron Beuoy, Jean P. Hall, Noelle K. Kurth, Kelsey S. Goddard

**Affiliations:** Institute for Health and Disability Policy Studies, University of Kansas, 1000 Sunnyside Ave., Room 1052, Lawrence, KS, 66045, USA

**Keywords:** Social connectedness, Transportation, Health, Disability

## Abstract

**Background:**

Transportation is an important resource for people to fully participate in their communities. People with disabilities who have access to reliable transportation report better social connectedness outcomes than those with less access. However, research has not yet examined how transportation access and other factors associated with social connectedness, such as self-rated health, influence social connectedness.

**Objective:**

The purpose of this study was to examine how access to transportation affects the association between self-rated health and social connectedness for people with disabilities.

**Methods:**

Moderated regressions were conducted using cross-sectional data from waves 2–4 of the National Survey on Health and Disability (NSHD) to examine the moderating effects of perceived transportation access on self-rated health and two dimensions of social connectedness: loneliness and social activity. The analysis focused on working-aged adults (18–64) with disabilities in the United States.

**Results:**

Transportation access moderated the relationship between self-rated health and loneliness but not social activity. Among people with disabilities who had access to reliable transportation, the magnitude of change between self-rated health and loneliness was stronger compared to those without reliable transportation.

**Conclusion:**

These findings highlight how transportation access plays a role in the relationship between health and social connectedness for people with disabilities. Policies that improve transportation access are needed to support greater social connectedness and better health outcomes for people with disabilities.

## 1. Introduction

People need transportation to fully participate in their communities and broader society (Jansuwan et al., 2013). Despite people with disabilities relying on public transportation more than the general public (Bezyak et al., 2017), they experience barriers to use these services, including inaccessibility due to physical pick-up and drop off locations, route complexities, and the amount of time it takes to travel (Sitter and Mitchell, 2020). People with disabilities have different travel needs than the general population (Bezyak et al., 2020), and failure to account for these patterns during public planning can worsen the transportation disadvantage (Park et al., 2023).

Ridesharing and paratransit are alternatives to fixed route transportation meant to help alleviate the barriers people with disabilities experience. Paratransit services, in particular, have been associated with a greater sense of independence (Desai et al., 2024), safety (Egger et al., 2022), and greater levels of social participation ability compared to those relying on family members or friends (Desai et al., 2023). However, the alternative transportation methods come with their own disadvantages. The cost of paratransit can be higher than fixed route methods and there are major gaps in coverage in largely rural states (Desai et al., 2024). Ride-sharing services have similar drawbacks to paratransit, in addition to often not providing accessible vehicles, and potentially refusing rides to customers who use service animals (Centers for Medicare and Medicaid Services, 2023). Medicaid and other insurance programs may cover the cost for non-emergency medical transportation services, but coverage for transportation to other activities, such as social events, is extremely limited (Heaps et al., 2021; Hinton and Diana, 2024; Lankford, 2024), ignoring the potential health benefits of increased opportunities for social connectedness.

Social connectedness is an umbrella term for the different ways people connect with each other socially (Holt-Lunstad et al., 2017) and is an important factor for the health and well-being of individuals (Holt-Lunstad, 2024). Two types of social connectedness studied previously are satisfaction with social participation and loneliness. Satisfaction with social participation is a person’s perceived satisfaction with their ability to participate in social activities (Repke and Ipsen, 2020). Loneliness is a lack of social connection and has been defined as the subjective feeling of being without desired relationships (Tanskanen and Anttila, 2016). Loneliness is associated with an increased risk of death (Repke and Ipsen, 2020; Tanskanen and Anttila, 2016), as well as mental and physical illness (Masi et al., 2011). In contrast, social participation has been linked to positive psychological health outcomes (Fiorillo et al., 2017) and a decreased risk for all-cause mortality (Holt-Lunstad et al., 2017). However, much of the existing literature on social participation and loneliness focuses on older adults(Repke and Ipsen, 2020), often overlooking the importance of these factors for the health of younger people with disabilities.

People with disabilities report lower levels of social interaction (Repke and Ipsen, 2020; Ipsen and Repke, 2022), higher levels of loneliness (Repke and Ipsen, 2020; Ipsen and Repke, 2022; Macdonald et al., 2018), and poorer self-rated health (Pagán, 2013; Shandra, 2018) compared to individuals without disabilities. Additionally, people with disabilities report lower incomes (Shandra, 2018; Ipsen et al., 2021), lower educational attainment (Shandra, 2018), and lower employment rates (Shandra, 2018). They are also more likely to live in geographically isolated areas (Repke and Ipsen, 2020) and to live alone (Emerson et al., 2021). These social inequalities have been linked to disparate health outcomes (Shandra, 2018), limited access to transportation (Bezyak et al., 2020), and reduced social connectedness (Hall et al., 2022) among people with disabilities. Taken together, poor health is associated with worse feelings of social connectedness(Hall et al., 2022), while access to transportation is associated with greater feelings of social connectedness (Ipsen and Repke, 2022), even after controlling for other sociodemographic and environmental factors. However, research has not yet examined how these three factors—health, transportation, and social connectedness—interrelate for people with disabilities. While poor health has been associated with worse feelings of social connectedness, access to transportation could serve as a resource for people with disabilities to connect with others. As a result, the relationship between health and social connectedness may change based on a person’s access to transportation.

The purpose of this study is to understand if access to transportation changes the relationship between self-rated health and two different measures of social connectedness for working-aged adults with disabilities, after controlling for sociodemographic and environmental factors, and disability indicators. An innovation of this study is its exploration of two distinct dimensions of social connectedness—social participation and loneliness—using moderated regressions. Our findings can inform how access to reliable transportation may help mitigate health inequalities, confirming and reinforcing the known importance of addressing transportation barriers for people with disabilities and suggesting specific policy changes. The sections that follow cover our methods, key results, and implications for policy and future research.

## 2. Methods

### 2.1. Data

For this study, we combined data from the second, third, and fourth waves of the National Survey on Health and Disability (NSHD), which were fielded October 2019 through January 2020; January through May 2021; and May through September 2022, respectively. The NSHD is a national online survey administered to adults across the United States with a broad range of disabilities over the age of 18 (University of Kansas Institute for Health and Disability Policy Studies, 2025). The purpose of the NSHD is to document the experiences working-aged adults with disabilities across 14 core domains. The core domains of interest for the current study were disability, health status, transportation, community participation, and demographics. Participants were recruited through disability-related organizations, national conferences, and Amazon Mechanical Turk (MTurk); more information about the domains, recruitment techniques, and data collection, can be found at https://ihdps.ku.edu/nshd. The current study focused on working aged adults with disabilities between the age of 18 and 64. If participants completed multiple waves of the NSHD, data from the most recent wave they completed was retained and the others were removed to facilitate between-person analyses.

### 2.2. Measures

#### 2.2.1. Dependent variables

The NSHD includes items pertaining to social connectedness. One item from the Patient-Reported Outcomes Measurement Information System (PROMIS; Hahn et al., 2010) was used to measure social activity: 1) “I am satisfied with my current level of social activity”; Response options were on a 0 (not at all) to 4 (very much) scale.

Loneliness was measured using the short form UCLA Loneliness Scale (Hughes et al., 2004). Participants responded to three items on a 1 (hardly ever) to 3 (often) scale: 1) “How often do you feel you lack companionship?”; 2) “How often do you feel left out?”; and 3) “How often do you feel isolated from others?” Responses were summed to create a composite score ranging from 3 to 9, with higher values indicating higher levels of perceived loneliness. The Cronbach’s alpha was 0.83, suggesting the three items are measuring the same underlying construct (Tavakol and Dennick, 2011) – loneliness.

#### 2.2.2. Independent variable

Self-reported health was the independent variable in this study. For general health, participants were asked, “In general, would you say your health is …” on a 0 (poor) to 4 (excellent) scale (Moriarty et al., 2003).

#### 2.2.3. Moderating variable

Perceived transportation access was used as the moderating variable in the current study. To measure transportation access, participants responded to the question, “How often do you have regular and reliable transportation to get to the places you need to go?” on a 0 (never) to 3 (all the time) scale; the item served as a general measure of transportation access.

#### 2.2.4. Covariates

Sociodemographic, environmental factors, and disability indicators were added as covariates because previous literature suggests they are related to health, transportation, and social connectedness, for people with disabilities; the covariates were coded to align with previous research (Repke and Ipsen, 2020; Ipsen and Repke, 2022; Macdonald et al., 2018; Pagán, 2013; Shandra, 2018; Ipsen et al., 2021; Emerson et al., 2021). Participants were asked demographic questions about yearly income (<138% federal poverty level, >138% federal poverty level), age (18–34, 35–64), gender (female, male, gender diverse), relationship status (single, not single), living arrangement (lives with someone, lives alone), educational status (high school or less, some college or more), employment status (employed, not employed), and race/ethnicity (white, non-white).

For disability indicator, participants were asked “Which ONE category would you use to describe your main disability or health condition?” Respondents could choose from seven options: physical/mobility; intellectual/cognitive; mental illness/psychiatric; chronic illness or disease; sensory; developmental; or neurological.

Participants were classified as living in a rural area if their county of residence had a population of less than 50,000 people based on county-level Rural Urban Commuting Area (RUCA; USDA ERS). More information about rural classifications can be found at: https://www.ers.usda.gov/topics/rural-economy-population/rural-classifications.

Finally, Survey year completed was included to account for between-year differences in the dependent variables. Accounting for between-year differences was important because COVID-19, and its restrictions, had an impact on health, transportation, and social connectedness (Borowski and Stathopoulos, 2023; Kutela et al., 2024; Ross, 2021) for some of the NSHD waves.

### 2.3. Data analysis

The purpose of the current study is to examine if the relationship between self-rated health and social connectedness changes based on a person’s access to transportation. A moderated regression (Baron and Kenny, 1986) is an appropriate method for understanding if the strength and/or direction of the relationship between an independent variable (IV; self-rated health) and dependent variable (DV; social connectedness) depends on a third variable, known as a moderator (transportation access). To test moderation, the product of the IV and moderator, called an interaction, is added to the regression model. A significant interaction suggests the relationship between the IV and DV is affected by the moderating variable. An additional strength of moderated regression is that covariates can be controlled by adding them to the model, and non-linear relationships between variables can be included. In the current study all variables were modeled as a linear relationship because, as described in 3. Results, scatter plots do not suggest a non-linear relationship between them.

Moderated multiple regressions were conducted to examine the interaction between self-rated health (IV) and transportation access (moderator) on loneliness and satisfaction with social activities, while controlling for sociodemographic, disability indicators, and environmental factors. Self-rated health and transportation access were treated as continuous in the regression models. Likert-type response data is commonly used in regression analyses as continuous; as long as the items have at least four response categories, the bias of using Likert-type items as continuous is minimized (Owuor, 2001).

Two separate moderated multiple regression models were run, one for each social connectedness dependent variable. For significant interaction terms, estimated marginal means (Searle et al., 1980) and simple slope plots were generated to illustrate how the relationship between general health and the dependent variable (DV) changed across each level of transportation access (0, 1, 2, and 3). Listwise deletion was used for incomplete cases, resulting in a sample of 4018 for the loneliness model and 3995 for the satisfaction with social activity model.

## 3. Results

### 3.1. Descriptive statistics

A majority of the respondents were female (62.9%), white (66.3%), between the ages of 35 and 64 (65.5%), employed (63.4%), had some college education or more (88%), and lived above 138% of the federal poverty level (68.3%). For self-categorized primary disability, 22.9% of respondents reported physical/mobility, 2.8% reported intellectual/cognitive, 24.9% reported mental illness/psychiatric, 28% reported a chronic illness of disease, 5.5% reported sensory, 4.6% reported developmental, and 11.2% reported neurological. Table 1 shows the descriptive statistics for all categorical variables included in the analyses. Table 2 shows the measures of central tendency, and minimum and maximum values for each continuous variable included in the analyses. Table 3 shows the bivariate correlations between the continuous variables.

### 3.2. Loneliness as the dependent variable

#### 3.2.1. Evaluating model assumptions

Scatter plots revealed a linear relationship between self-rated health, transportation access, the interaction between the two variables, and loneliness sum scores. No variance inflation factor (VIF) exceeded 10, suggesting there was no multicollinearity (Cohen et al., 2003). None of the studentized residuals exceeded ± 3 standard deviation, suggesting there were no outliers (Cohen et al., 2003). None of the Cook’s distance values were greater than one, suggesting none of the cases were influential (Cook and Weisberg, 1982). Using a hat value threshold of 2*22/4018 = 0.0109 (2p/n; Kutner et al., 2005), 182 cases were flagged as leverage points; 182 cases were removed from the data and the models were refitted. The refitted model’s fit, adjusted r-square, coefficients, and p-values, showed little change, so the original model was retained. A scatter plot of studentized residuals against predicted values showed equal error variances (homoscedasticity) (Weisburg, 2014). The studentized residuals were normally distributed (*M* = 0, *SD* = 1).

#### 3.2.2. Model results

The multiple regression model examining the interaction of general health and transportation on loneliness (Table 4) was statistically significant, *F*(22, 3880) = 46.40, *p* < .001, and explained 20.1 % of the variance in loneliness, *adj. R*^*2*^ = 0.201. The inclusion of the interaction significantly improved the model, *F*(1, 21) = 7.43, *p* < .001, and accounted for 0.2 % of the explained variation.

**Fig. 1. Figure 1.**
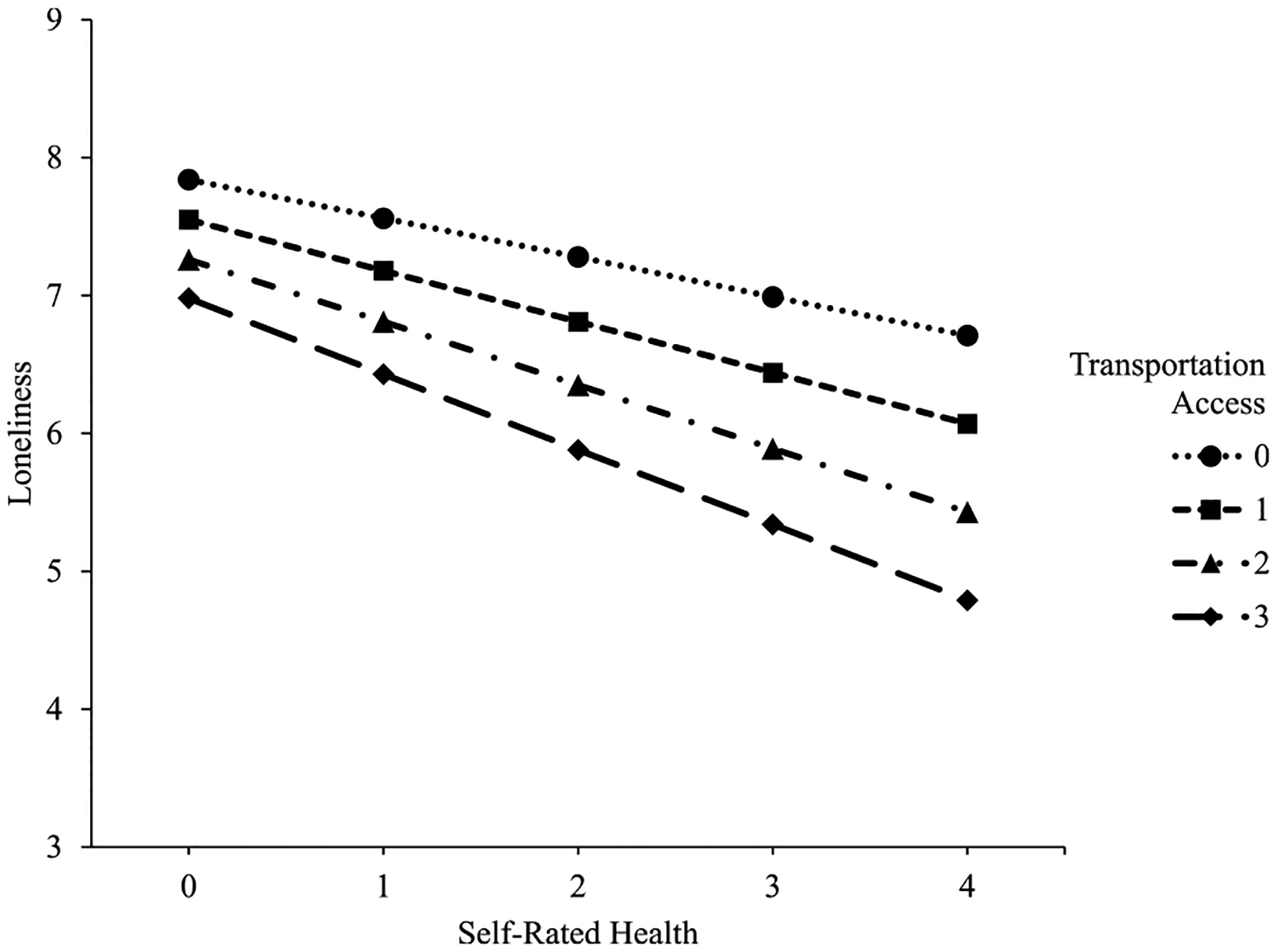
Predicted loneliness by self-rated health for every level of transportation access. Loneliness sum scores are represented on the y-axis and ranged from 3 to 9. Self-rated health, on the x-axis, ranged from 0 (poor) to 4 (excellent). Transportation access, represented by separate lines, ranged from 0 (never) to 3 (all the time).

There was a significant main effect of general health, *b* = −0.29, *se* = 0.08, *p* < .001, *95 % CI* [−0.46, −0.13], indicating feelings of loneliness decrease as general health increases. There was also a significant main effect of transportation access, *b* = −0.21, *se* = 0.09, *p* < .05, *95 % CI* [−0.38, −0.03], indicating feelings of loneliness decrease as access to transportation increases. The interaction between health and transportation access was statistically significant, *b* = −0.09, *se* = 0.03, *p* < .05, *95 % CI* [−0.02, −0.15]. The interaction indicates the relationship of general health on loneliness depended on transportation access. Estimated marginal means (Table 5) show the change in slope between general health and loneliness for every level of transportation access. For people with no access to regular and reliable transportation, a one-point increase in general health was associated with a 0.29 decrease in loneliness. The decrease became larger as access to regular and reliable transportation increased, capping at a 0.55 decrease for people who always had access to transportation. Even though loneliness decreased as perceived general health increased, the relationship between health and loneliness was stronger, represented by steeper slopes, for people with greater access to transportation, as illustrated in Fig. 1. Fig. 1 displays predicted loneliness by self-rated health for every level of transportation access while holding all other variables constant (simple slopes plot).

### 3.3. Social activity as the dependent variable

#### 3.3.1. Evaluating model assumptions

Scatter plots revealed a linear relationship between self-rated health, transportation access, the interaction between the two variables, and satisfaction with social activity scores. No variance inflation factor (VIF) exceeded 10, suggesting there was no multicollinearity (Cohen et al., 2003). Two cases had studentized residuals exceeding ± 3 standard deviation, suggesting they were outliers (Cohen et al., 2003). None of the Cook’s distance values were greater than one, suggesting none of the cases were influential (Cook and Weisberg, 1982). Using a hat value threshold of 2*22/3995 = 0.0110 (2p/n; Kutner et al., 2005), 179 cases were flagged as leverage points. The outlier and leverage cases were removed from the data and the models were refitted. The refitted model’s fit, adjusted r-square, coefficients, and p-values, showed little change, so the original model was retained. A scatter plot of studentized residuals against predicted values showed equal error variances (homoscedasticity) (Weisburg, 2014). The studentized residuals were normally distributed (*M* = 0, *SD* = 1).

#### 3.3.2. Model results

The multiple regression model examining the interaction of general health and transportation on satisfaction with social activity (Table 6) was statistically significant, *F*(22, 3858) = 41.49, *p* < .001, and explained 18.5 % of the variance in satisfaction with social activity, *adj. R*^*2*^ = 0.185. The inclusion of the interaction did not significantly improve the model, *F*(1, 1) = 1.32, *p* = .318.

There was a main effect of general health, *b* = 0.37, *se* = 0.06, *p* < .001, *95 % CI* [0.26, 0.48], indicating satisfaction with social activity increases as general health increases. There was no main effect of transportation access, *b* = 0.09, *se* = 0.06, *p* = .149, *95 % CI* [−0.03, 0.20], or an interaction between general health and transportation access, *b* = 0.02, *se* = 0.02, *p* = .314, *95 % CI* [−0.02, 0.07].

## 4. Discussion

This study aimed to examine the moderating effects of transportation access on health and two domains of social connectedness: Satisfaction with social activity and loneliness. Transportation access only moderated the relationship between self-rated health and loneliness. Self-rated health was a significant predictor for satisfaction with social activity and loneliness. Transportation access was only a significant predictor for loneliness.

The interaction between self-rated health and transportation access explained 0.2 % of the variance in loneliness. Even though the change in r-square was small, access to reliable transportation is practically important for managing health (Repke and Ipsen, 2020) and loneliness (Bezyak et al., 2020) for people with disabilities. The moderation effect suggests access to transportation plays a role in the relationship between health and feelings of being without desired relationships (loneliness). Holding health constant, people reported less loneliness as transportation access increased. A contributor to one’s sense of loneliness is their perceived autonomy and personal control (Ybarra and Chan, 2023). Access to transportation could serve as a method for people with disabilities to have personal control over their choices. This sense of control, in turn, could be used to find ways to reduce feelings of loneliness (Andrew and Meeks, 2018).

Self-rated health predicting all social connectedness variables is consistent with previous literature(Hall et al., 2022), suggesting a person’s health plays a role in their feelings of loneliness and satisfaction with social participation. Transportation access predicting loneliness is also consistent with previous literature (Ipsen and Repke, 2022). Much as with loneliness, transportation could be used as a method for attending social activities. The lack of a moderating effect of transportation on health and satisfaction with social participation, and a main effect of transportation on satisfaction with social activity could be due to the broad transportation question asked in the NSHD and some forms of social connectedness not requiring transportation.

Access to transportation for certain activities can affect some forms of social connectedness more than others. Previous research found a lack of transportation for social needs was a predictor of satisfaction with social activity, but a lack of transportation for daily needs was not (Ipsen and Repke, 2022). Barriers to transportation have the greatest effect on socializing or recreation (Bezyak et al., 2020), and two-thirds of people with disabilities say a lack of transportation negatively affects their social activity (Bascom and Christensen, 2017).

Not all forms of social connectedness require access to transportation. Considering people with disabilities travel less frequently, and a proportion of them do not leave home because of transportation difficulties (Bezyak et al., 2020), social media can help them socialize without needing to travel anywhere. Research suggests people with disabilities use social media to engage with individuals having similar disabilities and enhance positive relationships (Akhmedova et al., 2024), stay connected and share life events (Bayor et al., 2018), and experience feelings of social accessibility and acquire friendships (Farley, n.d.).

### 4.1. Future research

Future research should employ longitudinal analyses, which would provide insight on how self-rated health, transportation access, the interaction between them, and different forms of social connectedness change over time within individuals and how different factors can impact those relationships. As a result, policies targeting more impactful factors could be implemented to improve transportation access for people with disabilities.

Previous research has examined different types of transportation people with disabilities rely on (e.g., a car, public transportation, friends or family). Future research should analyze different types of primary transportation as a moderator to provide a nuanced understanding of how the relationship between health and social connectedness could change depending on the type of transportation a person relies on. Finally, more reliable measures of social activity, like the PROMIS Satisfaction with Participation in Social Roles and Activities (Desai et al., 2023), should be used to better capture people’s satisfaction with social activity.

### 4.2. Limitations

The NSHD does not ask if participants have access to transportation for specific activities (e.g., socializing or doing daily activities), or the type of transportation a person has access to and uses regularly (e.g., has a car, uses public transportation, relies on friends or family), so analyses cannot be conducted to understand how different forms of transportation access play a role in moderating health and social connectedness. The NSHD also does not ask participants about their online socialization behaviors, so the results cannot speak to whether virtual interactions play a role in social connectedness or if they are a suitable alternative when transportation is not accessible or available. Even though this study used health to predict social connectedness, conclusions about health causing social connectedness cannot be made. Research examining health and social connectedness for people with disabilities has found health can predict social connectedness, and vice versa. Whether health or social connectedness comes before the other is unclear, and disentangling this relationship was beyond the scope of the current study. Single item measures were used for transportation access and satisfaction with social activity, and they may not capture the constructs as well as multi-item scales. Finally, the sample was recruited online and was majority white, female, and educated, which is not representative of the disability population.

### 4.3. Implications

Understanding the relationships between access to transportation, social connectedness, and health among people with disabilities has important policy implications, such as enforcing ADA requirements for public transportation and ride-sharing services and expanding access to transportation for non-medical visits via Medicaid, Medicare, and private insurance. Paratransit initiatives like IRIS in Kansas City, Missouri, or RIDE in Boston, Massachusetts, employ app-based technologies, real time scheduling, dynamic routing for efficient routes, text services so riders can track arrivals and departures, and collaborations with ride share companies to provide subsidized trips instantly, and have been recognized as model paratransit services (Desai et al., 2024).

Adaptation of city-wide initiatives like IRIS in Kansas City, Missouri, or RIDE in Boston, Massachusetts, or at least some of the features the model paratransit services offer, could be effective methods for improving transportation access for people with disabilities in metropolitan areas. City-wide initiatives may not be applicable or feasible for smaller communities or rural areas. Per the Americans with disabilities Act of 1990 (Americans with disabilities Act of 1990), paratransit services must be offered in a three-quarter mile radius of fixed route transit, including rail stations. Policies that invest in building or expanding fixed routes systems, especially in rural areas, could improve transportation access because they would also require paratransit services to be established in a three-quarter mile radius.

Cost of paratransit services is a frequently reported barrier for people with disabilities (Egger et al., 2022). Currently, Medicaid transportation for non-medical reasons is limited to certain waiver programs and populations (Centers for Medicare and Medicaid Services, 2023; Hinton and Diana, 2024; Lynott). Similarly, Medicare transportation for non-medical reasons is limited to Advantage plan providers (Holt-Lunstad et al., 2017). Given the demonstrated health outcomes associated with lack of social connectedness (Holt-Lunstad et al., 2017), these limitations seem short-sighted and non-cost-effective. City wide initiatives and policies expanding Medicaid and Medicare to include subsidies for non-medical transportation for a greater number of programs and populations could remove barriers to transportation access and promote the health and well-being of people with disabilities.

## Figures and Tables

**Table 1:** Descriptives for Categorical variables

Variable	*n* ^a^	%
**Population Density**		
Urban	4159	85.3
Rural^b^	716	14.7
**Employment**		
Not Employed	1680	36.6
Employed^c^	2905	63.4
**Yearly Income**		
<138 % FPL^d^	1532	31.7
>138 % FPL^d^	3298	68.3
**Age Category**		
18–34	1693	34.5
35–64	3211	65.5
**Gender**		
Female	3072	62.9
Male	1341	27.5
Gender Diverse	471	9.6
**Relationship Status**		
Single	2547	52.9
Not Single	2266	47.1
**Living Arrangement**		
Lives with Someone	3606	77.3
Lives Alone	1087	22.7
**Survey Year**		
2020	1498	30.5
2021	1047	21.3
2022	2359	48.1
**Educational Status**		
High School or Less	584	12
Some College or More	4282	88
**Race/Ethnicity**		
White	3144	66.3
Non-White	1601	33.7
**Self-Categorized Disability** ^e^		
Physical/Mobility	1065	22.9
Intellectual/Cognitive	132	2.8
Mental Illness/Psychiatric	1162	24.9
Chronic Illness or Disease	1305	28
Sensory	257	5.5
Developmental	214	4.6
Neurological	523	11.2

a n’s are not equal due to missingness

b Population of less than 50,000 people based on county-level Rural Urban Commuting Area (RUCA) codes

c Work for pay, self-employed, or both

d FPL = federal poverty level

e Participants were asked to categorize their main disability or health condition into one of these seven options.

**Table 2:** Descriptives for Continuous Variables

Variable	*n* ^a^	*mean*	*median*	*SD*	*min*	*max*
Transportation Access	4892	2.43	3	0.81	0	3
General Health	4902	1.66	3	1.00	0	4
Loneliness^b^	4904	6.54	7	1.93	3	9
Social Activity	4867	1.5	1	1.27	0	4

Note:

* < .05;

** < .01;

*** < .001.

a n’s are not equal due to missingness

b Sum score of three items on the UCLA Loneliness Scale.

**Table 3:** Correlation Matrix for Continuous Variables

Variable	1	2	3	4
1) Transportation Access	1			
2) Self-Rated Health	0.15***	1		
3) Loneliness	−0.25***	−0.30***	1	
4) Social Activity	0.15***	0.38***	−0.62***	1

**Table 4:** Regression Analysis with Loneliness as the Dependent Variable^a^

Variable	*B*	*SE*	*t*	*p*	*95 % CI Lower*	*95 % CI Upper*
Intercept	8.60	0.26	33.12	<0.001	8.09	9.11
Self-Rated Health	−0.29	0.08	−3.47	<0.05	−0.46	−0.13
Transportation Access	−0.21	0.09	−2.34	<0.05	−0.38	−0.03
Self-Rated Health × Transportation Access	−0.09	0.03	−2.65	<0.01	−0.15	−0.02
Self-Categorized Disability (reference group: Physical/Mobility)^b^						
Intellectual/Cognitive	−0.03	0.18	−0.18	0.856	−0.38	0.32
Mental Illness/Psychiatric	0.58	0.08	7.12	<0.001	0.42	0.74
Chronic Illness or Disease	0.03	0.08	0.39	0.694	−0.13	0.19
Sensory	−0.37	0.13	−2.78	<0.01	−0.64	−0.11
Developmental	0.35	0.15	2.35	<0.05	0.06	0.64
Neurological	0.01	0.10	0.13	0.900	−0.19	0.21
Survey Year (reference year: 2020)						
2021	0.26	0.10	2.51	<0.05	0.06	0.47
2022	0.09	0.07	1.31	0.189	−0.04	0.22
Rural (reference group: Urban)^c^	−0.15	0.08	−1.88	0.060	−0.30	0.01
Employed (reference group: Unemployed)^d^	−0.36	0.06	−5.70	<0.001	−0.48	−0.23
Income <138 % FPL (reference group: Income >138 % FPL)^e^	−0.03	0.07	−0.40	0.686	−0.16	0.10
Age 35–64 (reference group: Age 18–34)	−0.15	0.06	−2.40	<0.05	−0.27	−0.03
Gender (reference group: Female)						
Male	0.09	0.06	1.34	0.182	−0.04	0.21
Gender Diverse	0.33	0.10	3.38	<0.01	0.14	0.53
Non-White (reference group: White or Caucasian)	−0.28	0.09	−3.18	<0.01	−0.45	−0.11
Not Single (reference group: Single)	−0.56	0.07	−8.45	<0.001	−0.69	−0.43
Lives Alone (reference group: Lives with someone)	0.28	0.08	3.63	<0.001	0.13	0.43
Some College or More (reference group: high school or less)	0.10	0.09	1.06	0.291	−0.08	0.27

a n = 4018.

b Participants were asked to categorize their main disability or health condition into one of these seven options.

c Population of less than 50,000 people based on county-level Rural Urban Commuting Area (RUCA) codes.

d Work for pay, self-employed, or both.

e FPL = federal poverty level.

**Table 5:** Estimated Marginal Means of Loneliness for Self-Rated Health and Levels of Transportation Access

Transportation Access	*Estimate*	*SE*	*z*	*p*	*95 % CI Lower*	*95 % CI Upper*
Never (0)	−0.29	0.08	−3.85	<0.001	−0.46	−0.13
Sometimes (1)	−0.38	0.05	−7.30	<0.001	−0.49	−0.27
Often (2)	−0.46	0.03	−14.34	<0.001	−0.53	−0.40
All the time (3)	−0.55	0.04	−15.29	<0.001	−0.62	−0.48

*Note*. Table 5 shows the change in slope between general health and loneliness for every level of transportation access.

**Table 6:** Regression Analysis with Satisfaction for Social Activity as the Dependent Variable^a^

Variable	*B*	*SE*	*t*	*p*	*95 % CI Lower*	*95 % CI Upper*
Intercept	0.24	0.17	1.37	0.170	−0.10	0.58
Self-Rated Health	0.37	0.06	6.55	<0.001	0.26	0.48
Transportation Access	0.09	0.06	1.44	0.149	−0.03	0.20
Self-Rated Health × Transportation Access	0.02	0.02	1.01	0.314	−0.02	0.06
Self-Categorized Disability (reference group: Physical/Mobility)^b^						
Intellectual/Cognitive	−0.02	0.12	−0.15	0.881	−0.25	0.22
Mental Illness/Psychiatric	−0.41	0.05	−7.54	<0.001	−0.52	−0.31
Chronic Illness or Disease	−0.08	0.05	−1.41	0.159	−0.18	0.03
Sensory	0.22	0.09	2.46	<0.05	0.05	0.40
Developmental	−0.22	0.10	−2.23	<0.05	−0.42	−0.03
Neurological	−0.11	0.07	−1.63	0.103	−0.25	0.02
Survey Year (reference year: 2020)						
2021	−0.16	0.07	−2.36	<0.05	−0.30	−0.03
2022	−0.10	0.05	−2.13	<0.05	−0.19	−0.01
Rural (reference group: Urban)^c^	0.08	0.05	1.53	0.126	−0.02	0.18
Employed (reference group: Unemployed)^d^	0.32	0.04	7.54	<0.001	0.23	0.40
Income <138 % FPL (reference group: Income >138 % FPL)^e^	0.05	0.04	1.11	0.266	−0.04	0.14
Age 35–64 (reference group: Age 18–34)	−0.11	0.04	−2.76	<0.01	−0.20	−0.03
Gender (reference group: Female)						
Male	0.04	0.04	0.89	0.371	−0.05	0.12
Gender Diverse	−0.02	0.07	−0.27	0.790	−0.15	0.11
Non-White (reference group: White or Caucasian)	0.12	0.06	2.00	<0.05	0.00	0.23
Not Single (reference group: Single)	0.15	0.04	3.28	<0.01	0.06	0.23
Lives Alone (reference group: Lives with someone)	−0.10	0.05	−1.86	0.062	−0.20	0.00
Some College or More (reference group: high school or less)	−0.12	0.06	−1.99	<0.05	−0.24	0.00

a n = 3995.

b Participants were asked to categorize their main disability or health condition into one of these seven options.

c Population of less than 50,000 people based on county-level Rural Urban Commuting Area (RUCA) codes.

d Work for pay, self-employed, or both.

e FPL = federal poverty level.

## Data Availability

Data will be made available on request.
